# One‐Year Follow‐Up of Clinical and Morphological Outcomes in Elite Athletes With Early‐Stage Lower Extremity Tendinopathy

**DOI:** 10.1002/ejsc.12303

**Published:** 2025-04-22

**Authors:** Marc Seidler, Rene B. Svensson, Christopher Meulengracht, Kasper Ø. Christensen, Christoffer Brushøj, Mathilde Kracht, Mikkel H. Hjortshoej, S. Peter Magnusson, Roald Bahr, Michael Kjær, Christian Couppé

**Affiliations:** ^1^ Department of Orthopaedic Surgery Institute of Sports Medicine Copenhagen Copenhagen University Hospital—Bispebjerg and Frederiksberg Copenhagen Denmark; ^2^ Faculty of Health and Medical Sciences Department of Clinical Medicine Center for Healthy Aging University of Copenhagen Copenhagen Denmark; ^3^ Department of Physical Therapy Musculoskeletal Rehabilitation Research Unit Copenhagen University Hospital—Bispebjerg and Frederiksberg Copenhagen Denmark; ^4^ Centre for Health and Rehabilitation University College Absalon Slagelse Denmark; ^5^ Department of Sports Medicine Oslo Sports Trauma Research Centre Norwegian School of Sports Sciences Oslo Norway

**Keywords:** early dectection, early tendinopathy, elite athletes, pain‐guided activity modification

## Abstract

Little is known about early tendinopathy in elite athletes. This study aimed to investigate changes in clinical and ultrasonography outcomes over 1 year and assess the prognostic values of these outcomes at baseline with respect to tendinopathy progression. Sixty‐two elite athletes (24 ± 5 years) with early phase (symptom duration < three months) Achilles or patellar tendinopathy (AT and PT) were examined at baseline and after one year. Pain‐guided activity modification was the only intervention. Clinical outcomes were assessed using Victorian Institute of Sports Assessment questionnaires (VISA) for function and symptoms, pain scores (1–10 numerical rating scale (NRS)) and ultrasound tendon morphology (thickness, echogenicity and power Doppler (PD) flow area). A linear mixed‐effects model analysed changes from baseline to 1 year. Athletes showed clinical improvements in VISA‐Achilles (baseline: 66 ± 5 vs. one‐year: 87 ± 2, 95% CI: 13–30, *p* < 0.0001 and effect size *d* = 3.8), VISA‐Patella (baseline: 69 ± 3 vs. one‐year: 86 ± 1, 95% CI: 10–26, *p* < 0.0001 and effect size *d* = 3.6) scores and most NRS pain scores (≥ 2 points). Tendinopathic Achilles tendons' peritendinous thickness was reduced (−0.79 mm, *p* = 0.0188 and effect size *d* = 0.5), whereas patellar tendons remained enlarged. For both AT and PT, lower baseline PD was associated with a greater reduction in thickness over time (*p* < 0.001) and higher baseline VISA scores were linked to greater increases in echo intensity over time (*p* = 0.0363). In conclusion, elite athletes with early phase AT and PT showed clinical improvement over 1 year, with morphological changes in tendinopathic Achilles tendons only. Lower baseline PD and better initial VISA scores represent a better prognosis for tendinopathy morphology and symptoms.

**Summary**

- Early Achilles and patellar tendinopathy symptoms improve clinically after 1 year similarly to recreational athletes.
- Pain‐guided activity modification may improve outcomes in elite athletes with early lower extremity tendinopathy.
- There were only modest morphological improvements in elite athletes with early lower extremity tendinopathy after 1 year.

## 1 Introduction

Tendon overuse injury (tendinopathy) is a considerable problem in elite athletes, and it has been estimated to have a prevalence as high as 20%–50% (Kujala et al. 2005; Lian et al. 2005; Docking et al. 2018; Florit et al. 2019). In affected athletes, the symptoms and reduction in performance can be long lasting, and some never return to their previous level of sports activity and often lead them to end their sports career (Cook et al. 1997; Kettunen et al. 2002; Visnes et al. 2024).

Chronic tendinopathy is characterised by fluid accumulation, thickening, structural changes and increased blood flow (Öhberg et al. 2001; Pingel et al. 2014), but it is unclear in what order these changes occur (Magnusson et al. 2010; Magnusson and Kjaer 2019; Malmgaard‐Clausen et al. 2022). Treatment of chronic tendinopathy (e.g., loading‐based rehabilitation) has improved in recent years, but little is known about the outcome of treatment initiated in the early phase (Malmgaard‐Clausen et al. 2022). A recent study in recreational athletes found that even in the early stage of tendinopathy (symptom duration < 3 months), tendon angiogenesis, size and tissue anabolic signalling were greater compared to the contralateral extremity (Tran et al. 2020). On the other hand, another study (Hanlon et al. 2023) found no impact of the symptom duration on measures of tendon health over 3, 6 or 12 months in recreational athletes with early tendinopathy.

A recent study focusing on the early phase of tendinopathy in recreational runners found that patients reporting symptoms for less than 1 month had greater clinical improvements after 12 weeks of physical rehabilitation than those with symptoms for two to 3 months at inclusion, indicating that early diagnosis, resistance training and load management may be beneficial (Malmgaard‐Clausen et al. 2021). However, it has been observed that loading‐based rehabilitation may not be as effective in elite athletes with chronic tendinopathy when sports activity is continued (Visnes et al. 2005; van Ark et al. 2016; Rieder et al. 2022). The early phase of tendinopathy has not been studied in elite athletes, who have a large incentive to recover quickly, and it remains unknown whether they behave similarly to recreational athletes regarding early pathogenesis of tendinopathy.

Several previous studies have demonstrated that asymptomatic elite athletes with tendon abnormalities on ultrasound imaging have an increased risk of developing future chronic tendinopathy, indicating that morphological changes of the tendon precede onset of symptoms (Cook, Khan, Kiss, Purdam, et al. 2000; Fredberg and Bolvig 2002; Visnes et al. 2015; McAuliffe et al. 2016; Cushman et al. 2025).

The primary aim of this study was to determine possible changes in clinical and imaging outcomes over the course of 1 year in elite athletes with early phase Achilles tendinopathy (AT) and patellar tendinopathy (PT). The secondary aim was to assess the prognostic value of the baseline clinical and ultrasonographic (US) outcomes on the progression of the injury 1 year later.

We hypothesised that the elite athletes with early phase tendinopathy would improve in both clinical and US outcomes after 1 year, and that a tendon thickness reduction would be associated with an improvement in clinical outcomes.

## 2 Material and Methods

### 2.1 Study Design and Overview

This study was an observational longitudinal study of early phase tendinopathy in adult elite athletes. The study consisted of two examinations of each participant, referred to as baseline and a follow‐up (1 year after baseline). Although a third examination was conducted at 3 months, its data are reported elsewhere (Meulengracht et al. 2024). To maintain clarity and avoid potential confusion, only the baseline and 1‐year follow‐up examinations are presented in this study. The examinations included a thorough clinical assessment, ultrasonography, functional tests and questionnaires about functional and clinical aspects of everyday life and sporting activities. Participants underwent a phone interview prior to the first examination. If participants had unilateral symptoms, both legs were examined and the symptomatic tendon was compared with the asymptomatic tendon (control). In bilateral cases, the most symptomatic side was chosen as the injured side (based on the clinical examination) and the contralateral side was considered the control.

All participants were at each examination given instructions on pain‐guided activity modification based on the pain‐monitoring model (Salaffi et al. 2004; Silbernagel et al. 2007; Silbernagel and Crossley 2015; Sprague et al. 2021) and were recommended not to routinely use NSAIDs or other pain‐relieving drugs (Malmgaard‐Clausen et al. 2021). The pain‐monitoring model is a guideline for athletes and is based on an eleven‐point (from zero (no pain) to ten (worst pain imaginable)) numerical rating scale (NRS) pain score, which is usable to guide whether the athlete should stop, modify or continue sports or physical activity. NRS pain score during and after training was allowed to reach five but should have decreased the following morning.

The study conformed to the guidelines of The Declaration of Helsinki, was approved by the Regional Ethics Committee and was preregistered on ClinicalTrials.gov in August 2018. All participants signed a declaration of informed consent at baseline.

### 2.2 Participants

Recruitment was achieved through collaboration with the Danish federations for badminton, handball and volleyball or through Team Denmark (governmental organisation that advances elite sports). The participants were also recruited from elite sports clubs in the urban area of Copenhagen and by staff members of Danish national teams (coaches, physiotherapists etc.). Staff members were contacted by mail or by phone and asked if they had any athletes with newly acquired lower extremity injuries believed to be tendinopathy. The athletes were then interviewed by phone and if tendinopathy seemed plausible, and they met the inclusion criteria, they were included in the study and further assessed clinically.

During the period of August 16, 2018 to November 1, 2021, a total of 65 adult (> 18 years old) elite athletes (competing at the highest national level in their age group in their given sport) were recruited. The inclusion criteria were clinical signs of tendinopathy of their Achilles or patellar tendon lasting less than 3 months, defined as tendon pain related to sports activity, relevant pain during palpation or functional test (see description of clinical outcomes) in the affected tendon compared to the asymptomatic or less affected extremity (Tran et al. 2020). Athletes with early phase tendinopathy were divided into 3 subgroups: (a) symptoms for < 1 month (T1), (b) 1–2 months (T2) or 2–3 months (T3) (Tran et al. 2020).

### 2.3 Exclusion, Loss to Follow‐Up and Missing Data

**FIGURE 1:**
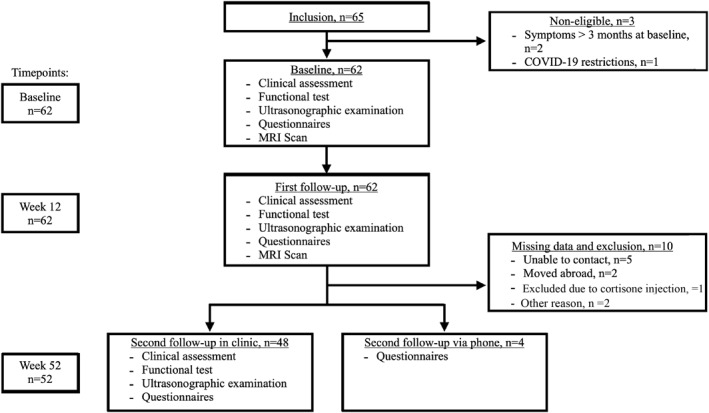
Study flowchart. Flowchart of the study population listing inclusion, exclusion, missing data and assessment at baseline, first follow‐up (3 months) and second follow‐up (1‐year). Four of the second follow‐ups were done via phone‐interviews, because the participants could not find a suitable day for clinical and ultrasonographic examination.

Participants were excluded if they had a recent infection or had received any kind of injection or surgery to their Achilles or patellar tendon or had a prior diagnosis of tendinopathy in the affected tendon. In total, 52 participants were included in the assessment of clinical outcomes and 48 participants were included in the assessment of the US outcomes. See Figure 1 for detailed information.

### 2.4 Clinical Outcomes

Self‐reported pain intensity was assessed on the eleven‐point NRS from zero (no pain) to ten (worst pain imaginable) and was registered for the following situations: (1) during sports or physical activity/training, (2) after sports or physical activity/training, (3) during resting periods between sports or physical activity/training, (4) in the morning and (5) maximum pain experienced in the last week (Salaffi et al. 2004; Silbernagel et al. 2007; Silbernagel and Crossley 2015; Sprague et al. 2021). In addition, participants rated their pain using the same NRS during (6) a functional test. Participants with PT performed a single‐leg decline squat on a board with a 25‐degree decline (Purdam et al. 2003). They were asked to perform the exercise twice, once to become familiar with the exercise and another where they had to rate their pain. Participants with AT performed 25 one‐legged jumps (Silbernagel et al. 2006) and similarly rated their pain. The functional tests were performed after the ultrasound examination to avoid any interference with the recordings (Pingel et al. 2013; Syha et al. 2014).

To quantify the severity of tendinopathy, we employed the Victorian Institute of Sports Assessment (VISA) questionnaires (Visentini et al. 1998; Robinson et al. 2001; Iversen et al. 2016). Participants completed the Danish version (Iversen et al. 2016) of the VISA‐Patella (VISA‐P) or VISA‐Achilles (VISA‐A) questionnaire via REDCap hosted by the Capital Region of Denmark (RegionH) (Harris et al. 2009, 2019). The VISA questionnaires consist of eight questions and the VISA score indicates severity of the symptoms and limitations of function and activity caused by the tendon injury with a maximum score of 100 indicating an asymptomatic and fully performing individual.

### 2.5 Ultrasonography

Participants were told to refrain from training or hard work 24 h prior to their examination. All imaging was performed twice, with the examiner lifting the probe off the skin in between recordings.

**FIGURE 2:**
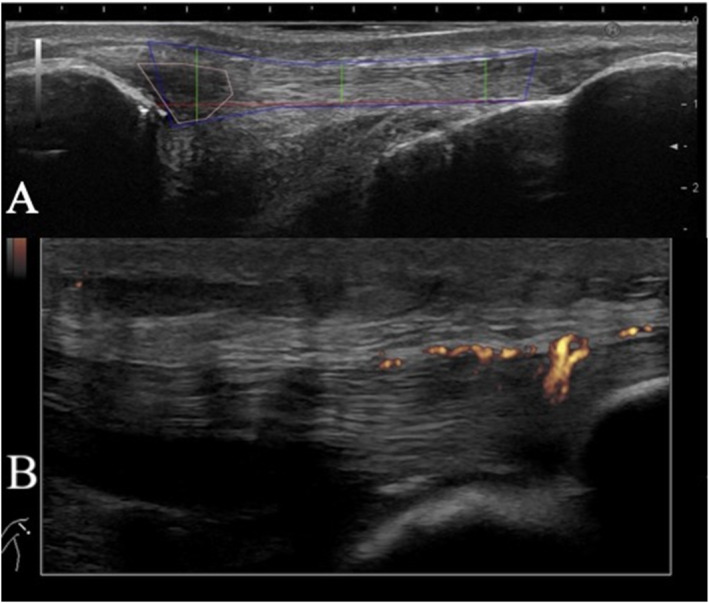
Ultrasonographic evaluation. Sagittal ultrasonographic images of a patellar tendon with left on the image being proximal and right being distal. The upper image (A) visualises the measurements of the thickness and hypoechoic region of the patellar tendon. Firstly, the distance from apex patella to the insertion of the tendon onto the tibial tuberosity was measured (red line); secondly, the anterior–posterior thickness was measured 0.5 cm distal from apex patella at the midpoint and 0.5 cm proximal from the tibial insertion (green lines). Finally, echointensity was measured in the whole tendon (blue outline) and in the hypoechoic region (pink outline). The lower image (B) is an example of the Doppler flow in the patellar tendon and in the peritendinous tissue.

The same US machine and software settings were used for all examinations (See supplementary materials). US findings were imported to Fiji/ImageJ (Schindelin et al. 2012) (Version 1.53, National Institute of Health, USA) for quantitative analyses. The specific measuring site and method for quantification were as previously described (Fredberg et al. 2008; Tran et al. 2020; Sigurðsson et al. 2022), see Figure 2 for further information. The greatest of the anterior–posterior (AP) thicknesses from the two recorded images was used for further statistical analysis.

The whole tendon as well as the visibly apparent hypoechoic region within the tendon were marked (see Figure 2). A macro was used to measure the mean echo intensity (from 0: black to 255: white) within both regions (Hjortshoej et al. 2024). All measurements were performed by the same investigator (blinded to participant pain scores and timepoints). The examiner was an experienced sports physician or well‐trained medical student, and all measurements were double‐checked by an experienced senior researcher in sports medicine. Outliers in the measurements were examined, and if caused by poor image quality, the image was excluded. Hypoechogenicity was analysed via two different methods for any difference over time (Hjortshoej et al. 2024). The first method compared the mean echogenicity for pixels in the hypoechoic region of the injured tendon relative to the corresponding region in the control tendon calculated as a percentage. The second method compared the mean echogenicity for pixels in the hypoechoic region of the injured tendon relative to the mean score of all pixels in the same tendon calculated as a percentage.

In the patellar tendon, AP thickness was measured at three different places referred to as proximal thickness (0.5 cm distal to the apex patella), distal thickness (0.5 cm proximal to the tibial insertion) and mid thickness (midway between these two previous points) (Fredberg et al. 2008; Tran et al. 2020; Hjortshoej et al. 2024).

In the Achilles tendon, AP thickness was measured at four different places referred to as proximal thickness (0.5 cm distal to the soleus insertion on the Achilles tendon), distal thickness (0.5 cm proximal to the Achilles tendon insertion on the calcaneus), max thickness (manually defined by the examiner) and peritendinous thickness (the point of max thickness including also peritendinous tissue) (Fredberg et al. 2008; Tran et al. 2020; Sigurðsson et al. 2022). Unless a specific site is indicated, the reported tendon thickness refers to the maximum thickness across the measurement sites (excluding peritendinous tissue).

### 2.6 Power Doppler Assessment

PD imaging for Achilles tendons was conducted with participants in a prone position, allowing their feet and ankles to hang relaxed and free from the bed. For patellar tendons, PD imaging was performed while participants lay supine with their knees stretched and relaxed. The transducer was placed sagitally at a 90‐degree angle and moved medially to laterally to locate the maximum power Doppler (PD) signal, while applying as little transducer pressure as possible. At this location, two four‐second sinae loops were recorded (uncompressed AVI files each containing 16 frames) see Figure 2. The ultrasound images were imported to Fiji/ImageJ for quantitative analysis. A custom macro was used to determine the frame containing the largest area of PD activity in each series (Eliasson et al. 2016; Hjortshoej et al. 2024). PD activity was only included if localised within the tendon and any noise signal was excluded. Before analysis, an experienced physician manually erased PD activity outside the tendons from the frames and removed any frames that contained noise signals due to probe movement. Noise can be distinguished from the vascular PD signal based on the consistency of the location, pattern and pulsation of the Doppler signal. US equipment settings and examination specifications can be seen in supplementary materials.

### 2.7 Statistical Analysis

The participants were grouped according to the location of injury (AT or PT). Further subgroups were defined depending on their initial symptom duration at baseline (T1: symptoms < 1 month, T2: symptoms 1–2 months and T3: symptoms 2–3 months). For PD analysis, the image with the largest PD area was used for further analysis. Analyses were performed by the same blinded investigator. We used the total score of PD for the analyses and analysed and compared potential differences over time in all groups. Differences in VISA and NRS pain scores between groups as well as differences over time within groups were analysed.

For all clinical and US outcomes, we used a two‐way ANOVA mixed‐effect analysis, with timepoint (baseline and 1 year, repeated) and symptom duration group (T1, T2 or T3, not repeated) as factors (Graphpad PRISM version 9.5, Boston, USA). *p*‐values < 0.05 were considered statistically significant and *p*‐values < 0.1 were considered to represent a trend. AT and PT were analysed separately. When there was no interaction or main effect of the duration group, the term ‘all’ is used to indicate effects across the duration groups. For baseline US outcomes, we also compared the symptomatic to the contralateral tendon using a paired *t*‐test. Values for the difference between baseline and second follow‐up are referred to as ‘delta’.

To investigate the prognostic value of the baseline US and clinical status on the progression of the same US and clinical outcomes, we used backwards elimination via a linear mixed regression model (SAS version 9.2, Cary, USA). The parameters we used as predictors in the model were the initial symptom duration group (T1, T2 or T3), injury site (AT or PT), baseline PD, baseline thickness, baseline echo intensity of the hypoechoic region relative to the whole tendon on the injured side, baseline NRS pain score when performing the functional test and baseline VISA score. The outcomes we tried to predict were the change from baseline to 1 year in the same parameters that were used as predictors (PD, thickness, echo intensity, NRS pain score in functional test and VISA score). Note that the baseline value of a given parameter was included in the model predicting the change in the same parameter, this is to account for the fact that the magnitude of change can depend on the baseline value, for example, an NRS score of two at baseline can at most decrease by two points.

## 3 Results

### 3.1 Participants

A total of 65 participants were enroled in the study from 2018 to 2021. 62 of these participants were included at baseline (men 66.1%, age: 24 ± 5.3 years and BMI: 23.7 ± 3.7 kg/m^2^); 52% with Achilles tendinopathy, 45% with their dominant leg injured and 22% with bilateral tendinopathy. The most common sports were volleyball (19%) and handball (18%). There were no significant differences in baseline characteristics between the groups (T1, T2 and T3). Further characteristics of subgroups can be found in Table 1.

**TABLE 1:** Baseline characteristics.

Characteristic	Total (*n* = 62)	T1 (*n* = 19)	T2 (*n* = 23)	T3 (*n* = 20)
Sex, *n* (%)				
Male	41 (66.1)	13 (68.4)	14 (60.9)	14 (70)
Female	21 (33.9)	6 (31.6)	9 (39.1)	6 (30)
Age, years	24.0 ± 5.3	25.1 ± 6.9	23.1 ± 4.0	24 ± 5.1
Height, cm	182.1 ± 8.5	183.3 ± 9.4	179.9 ± 8.8	183.4 ± 7
Weight, kg	78.9 ± 16	82.2 ± 9.4	74.1 ± 8.8	81.2 ± 22.4
Body mass index, kg/m^2^	23.7 ± 3.7	24.4 ± 2.4	22.8 ± 1.9	24 ± 5.7
Waist circumference, cm	81.4 ± 2.7	83.8 ± 7.0	78.4 ± 5.7	81.9 ± 13
Symptomatic tendon, *n* (%)				
Achilles	32 (51.6)	8 (42.1)	15 (65.2)	9 (45)
Patellar	30 (48.4)	11 (57.9)	8 (34.8)	11 (55)
Dominant leg injured, *n* (%)^a^				
Yes	27 (45)	7 (41.2)	11 (47.8)	9 (45)
No	20 (33.3)	7 (41.2)	7 (30.4)	6 (30)
Bilateral cases, *n* (%)	13 (21.7)	3 (17.6)	5 (21.7)	5 (25)
Sport, *n* (%)				
Badminton	10 (16.1)	2 (10.5)	6 (26.1)	2 (10)
Handball	11 (17.7)	3 (15.8)	5 (21.7)	3 (15)
Volleyball	12 (19.4)	3 (15.8)	4 (17.4)	5 (25)
Running	10 (16.1)	4 (21.1)	3 (13.0)	3 (15)
Athletics	5 (8.1)	1 (5.3)	1 (4.3)	2 (10)
Other^b^	14 (22.6)	6 (31.6)	4 (17.4)	5 (25)
Training per week before injury, hours	13.4 ± 4.7	14.4 ± 5.6	13.4 ± 4.4	12.5 ± 4.2

*Note:* Values are expressed as mean ± standard deviation unless otherwise specified. There were no significant differences between the groups, when using a one‐way ANOVA test and neither when using a Fischer's exact test to test for difference in sports. T1: Baseline symptom duration < 1 month. T2: Baseline symptom duration 1–2 months. T3: Baseline symptom duration 2–3 months.

^a^ If subjects had unknown leg dominance, they were excluded from the dominant leg descriptions and analyses in the table.

^b^ Other sports include football, gymnastics, weightlifting, sailing, karate, fencing, horse riding and triathlon.

### 3.2 Clinical Outcomes

The initial symptom duration (the three subgroups) had no significant influence on any of the pain or VISA scores, but there were main effects of time (see Tables 2 and 3 for detailed information).

**TABLE 2:** Clinical outcomes for Achilles tendinopathies.

Achilles	Baseline means ± SEM	52 weeks means ± SEM	Delta	95% CI	Delta (%)	*p*‐value
All NRS pain during sport/training	4.22 ± 0.45	0.91 ± 0.14	3.30	2.24 to 4.35	78.15	< 0.0001^a^
T1	3.88 ± 0.74	1.17 ± 0.83	2.68	0.12 to 5.23	69.03	0.0386^a^
T2	3.67 ± 0.58	0.69 ± 0.40	2.96	1.17 to 4.75	80.65	0.0009^a^
T3	5.11 ± 0.61	0.86 ± 0.46	4.26	1.87 to 6.64	83.25	0.0004^a^
All NRS pain after sport/training	3.00 ± 0.12	0.62 ± 0.11	2.32	1.30 to 3.34	77.35	0.0001^a^
T1	2.75 ± 0.75	0.83 ± 0.65	1.83	−0.66 to 4.32	66.55	0.1987
T2	3.13 ± 0.69	0.46 ± 0.31	2.61	0.88 to 4.35	83.36	0.0023^a^
T3	3.11 ± 0.72	0.57 ± 0.43	2.52	0.20 to 4.84	80.87	0.0306^a^
All NRS pain when at rest	0.59 ± 0.16	0.20 ± 0.14	0.39	−0.20 to 0.98	65.74	0.1938
T1	0.88 ± 0.48	0.00 ± 0.00	0.88	−0.54 to 2.29	100.00	0.3477
T2	0.33 ± 0.21	0.46 ± 0.46	−0.13	−1.12 to 0.86	−38.46	0.9846
T3	0.56 ± 0.18	0.14 ± 0.14	0.41	−0.90 to 1.73	74.29	0.8278
All NRS pain in the morning	2.85 ± 0.42	0.70 ± 0.08	2.09	1.15 to 3.04	73.40	0.0001^a^
T1	2.63 ± 0.80	0.83 ± 0.48	1.89	−0.42 to 4.19	71.89	0.1324
T2	2.27 ± 0.57	0.69 ± 0.46	1.51	−0.10 to 3.11	66.49	0.0699
T3	3.67 ± 0.71	0.57 ± 0.30	2.89	0.74 to 5.04	78.82	0.0063^a^
All NRS pain weekly max	5.08 ± 0.40	1.30 ± 0.18	3.75	2.38 to 5.11	73.74	< 0.0001^a^
T1	4.63 ± 0.89	1.67 ± 1.17	2.94	−0.32 to 6.20	63.61	0.0853
T2	4.73 ± 0.72	1.08 ± 0.46	3.63	1.35 to 5.91	76.73	0.0014^a^
T3	5.89 ± 0.87	1.17 ± 0.60	4.67	1.49 to 7.86	79.32	0.0030^a^
All NRS pain functional test (injured side)	3.07 ± 0.23	0.84 ± 0.50	2.22	0.96 to 3.48	72.26	0.0015^a^
T1	3.13 ± 0.83	1.83 ± 1.14	1.36	−1.61 to 4.33	43.49	0.5731
T2	2.64 ± 0.71	0.36 ± 0.36	2.20	−0.01 to 4.41	83.21	0.0515
T3	3.44 ± 0.87	0.33 ± 0.21	3.10	0.19 to 6.00	89.91	0.0346*
All NRS pain functional test (uninjured side)	0.80 ± 0.18	0.66 ± 0.09	0.15	−0.62 to 0.91	18.47	0.6906
T1	1.13 ± 0.67	0.67 ± 0.33	0.43	−1.37 to 2.24	38.49	0.9019
T2	0.50 ± 0.20	0.82 ± 0.55	−0.35	−1.69 to 0.10	−69.16	0.8823
T3	0.78 ± 0.52	0.50 ± 0.50	0.36	−1.41 to 2.13	45.85	0.9386
All VISA‐A score	65.60 ± 4.65	87.37 ± 2.19	21.63	−30.16 to −13.11	−32.97	< 0.0001^a^
T1	58.13 ± 3.91	83.17 ± 11.70	24.97	−45.79 to −4.16	−42.96	0.0154^a^
T2	74.13 ± 3.83	88.36 ± 4.28	14.22	−28.41 to −0.04	−19.18	0.0493^a^
T3	64.56 ± 5.38	90.57 ± 4.37	25.70	−45.10 to −6.31	−39.81	0.0070^a^

*Note:* Means are of the raw data (including missing values), whereas delta values and their associated statistics are based on the predicted means of the statistical model (accounting for missing values).

Abbreviations: All, refers to T1, T2 and T3 combined; CI, confidence interval; NRS, numerical rating scale (0–10); SEM, standard error of mean; T1, initial symptom duration < 1 month; T2, initial symptom duration 1–2 months; T3, initial symptom duration 2–3 months and VISA‐A, Victorian Institute of Sports Assessment questionnaire—Achilles.

^a^ Statistical significance (*p* < 0.05).

**TABLE 3:** Clinical outcomes for patellar tendinopathies.

Patellar	Baseline means ± SEM	52 weeks mean ± SEM	Delta	95% CI	Delta (%)	*p*‐value
All NRS pain during sport/training	3.93 ± 0.16	1.54 ± 0.30	2.39	1.21 to 3.57	60.68	0.0004^a^
T1	3.82 ± 0.57	1.29 ± 0.71	2.54	−0.08 to 5.16	66.47	0.0596
T2	4.25 ± 0.88	2.13 ± 1.11	2.13	−0.54 to 4.79	50.00	0.1469
T3	3.73 ± 0.66	1.20 ± 0.55	2.49	0.16 to 4.83	66.91	0.0343^a^
All NRS pain after sport/training	4.10 ± 0.61	1.91 ± 0.36	2.17	0.10 to 3.34	52.90	0.0009^a^
T1	4.18 ± 0.72	2.14 ± 1.06	1.99	−0.64 to 4.62	47.54	0.1785
T2	5.13 ± 0.61	2.38 ± 1.07	2.75	0.11 to 5.39	53.66	0.0391^a^
T3	3.00 ± 0.77	1.20 ± 0.57	1.77	−0.55 to 4.09	59.07	0.1722
All NRS pain when at rest	0.97 ± 0.30	0.37 ± 0.06	0.61	−0.20 to 1.42	62.53	0.1322
T1	1.36 ± 0.43	0.29 ± 0.29	1.09	−0.68 to 2.87	80.08	0.3299
T2	0.38 ± 0.26	0.50 ± 0.50	−0.13	−1.94 to 1.69	−33.33	0.9973
T3	1.18 ± 0.71	0.33 ± 0.24	0.86	−0.78 to 2.50	72.68	0.4666
All NRS pain in the morning	1.49 ± 0.48	0.70 ± 0.11	0.80	0.03 to 1.57	53.73	0.0412^a^
T1	0.73 ± 0.33	0.71 ± 0.71	0.01	−1.69 to 1.71	1.46	> 0.9999
T2	2.38 ± 0.82	0.88 ± 0.30	1.50	−0.23 to 3.23	63.16	0.1018
T3	1.36 ± 0.43	0.50 ± 0.34	0.89	−0.63 to 2.40	65.18	0.3724
All NRS pain weekly max	5.39 ± 0.32	2.27 ± 0.15	3.12	1.81 to 4.43	57.90	< 0.0001^a^
T1	4.91 ± 0.62	2.14 ± 0.80	2.77	−0.04 to 5.57	56.34	0.0547
T2	6.00 ± 0.65	2.57 ± 1.00	3.43	0.42 to 6.44	57.15	0.0206^a^
T3	5.27 ± 0.86	2.10 ± 0.72	3.17	0.64 to 5.71	60.18	0.0099^a^
All NRS pain functional test (injured side)	3.13 ± 0.57	1.13 ± 0.77	2.00	1.02 to 2.99	64.05	0.0002^a^
T1	3.64 ± 0.80	2.67 ± 0.71	0.97	−1.22 to 3.16	26.67	0.6257
T2	3.75 ± 0.59	0.38 ± 0.18	3.38	1.21 to 5.54	90.00	0.001^a^
T3	2.00 ± 0.56	0.33 ± 0.17	1.67	−0.28 to 3.61	83.35	0.1124
All NRS pain functional test (uninjured side)	0.83 ± 0.36	0.53 ± 0.12	0.32	−0.39 to 1.04	38.78	0.3558
T1	1.55 ± 0.61	0.50 ± 0.50	1.07	−0.57 to 2.71	69.49	0.2788
T2	0.50 ± 0.38	0.75 ± 0.49	−0.25	−1.81 to 1.31	−50.00	0.9675
T3	0.45 ± 0.37	0.33 ± 0.24	0.15	−1.27 to 1.57	31.97	0.9911
All VISA‐P score	68.53 ± 2.54	85.98 ± 1.43	18.01	−25.80 to −10.22	26.28	< 0.0001^a^
T1	69.09 ± 4.24	83.14 ± 5.47	15.35	−32.51 to 1.81	22.22	0.0888
T2	63.88 ± 4.61	87.13 ± 5.43	23.25	−40.70 to −5.80	36.40	0.0071^a^
T3	72.64 ± 5.47	87.67 ± 3.54	15.44	−31.25 to 0.37	21.26	0.0569

*Note:* Means are of the raw data (including missing values), whereas delta values and their associated statistics are based on the predicted means of the statistical model (accounting for missing values).

Abbreviations: All, refers to T1, T2 and T3 combined; CI, confidence interval; NRS, numerical rating scale (0–10); SEM, standard error of mean; T1, initial symptom duration < 1 month; T2, initial symptom duration 1–2 months; T3, initial symptom duration 2–3 months and VISA‐A, Victorian Institute of Sports Assessment questionnaire—Achilles.

^a^ Statistical significance (*p* < 0.05).

After 1 year, both the AT and the PT groups showed significant improvement in most NRS pain scores, but none of the groups or subgroups had any significant change in NRS pain score on the injured leg at rest or when performing the functional test on the uninjured leg.

**FIGURE 3:**
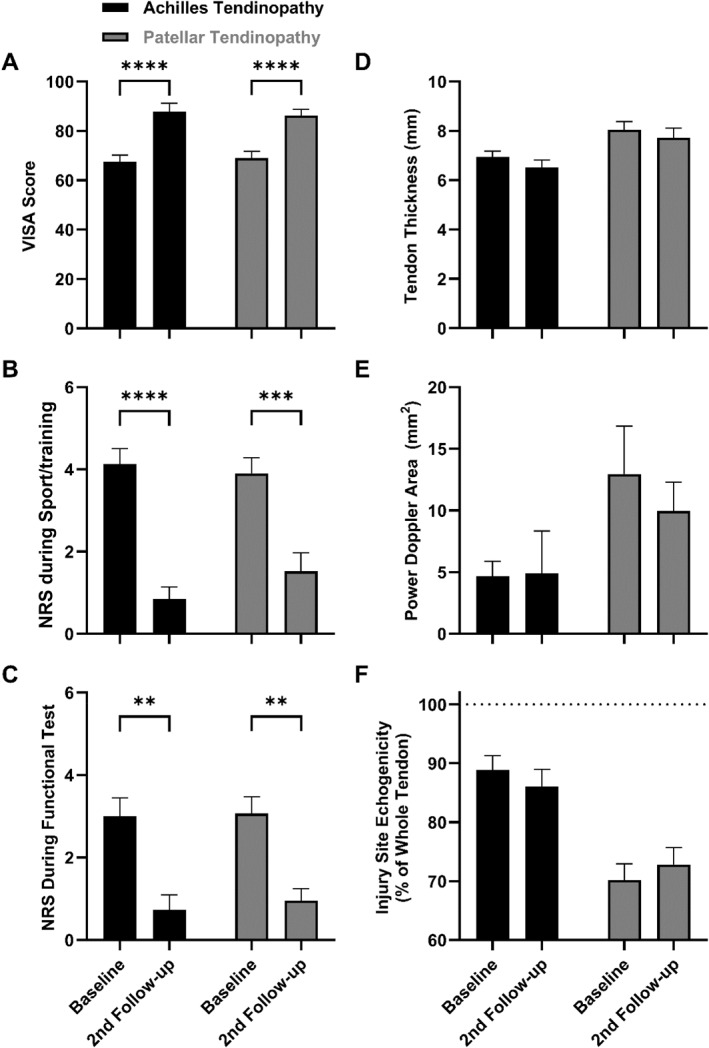
Clinical and ultrasonographic outcomes. (A–C) Clinical outcomes, pain on numerical rating scale ranging from 0 to 10 (NRS) and Victorian Institute of Sports Assessment questionnaire score (VISA score) as means with standard error. For the Achilles and patellar tendinopathy groups NRS during training, NRS during the functional test on the injured extremity and the VISA score all significantly improved from baseline to the second follow‐up (1‐year after baseline). * indicates level of significance: *(*p* < 0.05), **(*p* < 0.01), ***(*p* < 0.001) and ****(*p* < 0.0001). (D–F) Ultrasonographic outcomes, anterior–posterior tendon thickness (mm), power Doppler area (mm^2^) and hypoechogenicity (percentage of the whole tendons echogenicity) as means with standard error. For the Achilles tendinopathy group, tendon thickness tended to be reduced (−0.44 mm and *p* = 0.089) at the 2nd follow‐up after 1 year.

For AT, all groups (All AT, T1, T2 and T3) showed significant improvement in VISA scores after 1 year (All AT: delta 21.63 and *p* < 0.0001) see Figure 3. For the PT groups, there was only a significant improvement in the VISA score for the whole group and the T2 subgroup. (All PT: delta 18.01, *p* < 0.0001).

### 3.3 Ultrasonography

All results of ultrasonographic measurements can be seen in s Supporting Information S1: Tables S4 and S5. Similar to the clinical outcomes, the initial symptom duration had no significant influence on any of the morphological outcomes. AP thickness for PT (delta 17.4% and *p* = 0.0024) and AP thickness for AT (delta 14.7% and *p* = 0.0037) were increased compared to the contralateral leg at baseline. In the PT group at baseline, the entire injured tendon showed less echogenicity compared to the entire contralateral tendon (delta −6.64% and *p* = 0.0009) as well as less echogenicity in the hypoechoic region relative to the same region in the contralateral tendon (delta −10.6% and *p* = 0.0266).

The AT group showed a trend towards reduced distal thickness (delta −0.44 mm and *p* < 0.089) after 1 year. When including the peritenon, the AT group had a significant decrease in tendon thickness (delta −0.79 mm and *p* = 0.0188) after 1 year. Neither the PT group nor any of the PT subgroups showed any significant change in tendon thickness over time.

None of the groups or subgroups had any significant change in PD over time. Neither of the two methods we used to test for hypoechogenicity demonstrated any significant change over time in any of the groups or subgroups.

### 3.4 Prognostic Values

See Supporting Information S1: Table S6 for detailed information about the multiple regression of changes in outcomes from baseline to 1‐year follow‐up. Tendon type (Achilles and patellar) was not a significant predictor of change for any of the outcomes. Change in PD was not predicted by any parameters. Change in thickness over 52 weeks was predicted to be greater (i.e., an increase or lesser decrease in thickness) if baseline PD was greater (parameter estimate 0.083 mm/mm^2^ *p* = 0.0004) and to be lesser if baseline thickness was greater (parameter estimate −0.732 mm/mm and *p* < 0.0001). Change in echo intensity was predicted to be greater if baseline VISA score was greater (parameter estimate 0.254%/point and *p* = 0.0363) and to be lesser if echo intensity at baseline was greater (parameter estimate −0.396%/% and *p* = 0.0016). Change in the NRS pain score during the functional test was predicted to be lesser if the initial symptom duration was greater (parameter estimate −0.809 point/month and *p* = 0.0082) and if the NRS pain score during the functional test at baseline was greater (parameter estimate −0.898 point/point and *p* < 0.0001). Change in the VISA score was predicted to be lesser if the VISA score at baseline was greater (parameter estimate −0.712 point/point and *p* < 0.0001).

## 4 Discussion

The main findings were that symptoms and function improved in elite athletes after 1 year in both the AT and PT groups. Both the VISA and NRS pain scores improved in both groups independent of the prior symptom duration. Interestingly, only moderate changes in the morphological parameters were observed over the course of 1 year. Achilles peritenon thickness was reduced and Achilles tendon thickness tended to be reduced, whereas the patellar tendon thickness did not change over time. Furthermore, lower baseline PD results were linked with increases in echo intensity after 1 year within the pathological region of the affected tendon across both tendon types.

These data suggest that elite athletes with either early AT or PT exhibit clinical improvement after 1 year, and in cases with early AT, peritendinous tissue swelling can be reduced after 1 year. In addition, longer symptom duration and less severe outcomes at baseline (lower PD and higher VISA score) appear to predict a better prognosis for both morphology and symptoms after 1 year. Other studies have found that recreational athletes with symptom duration of less than 1 month did not differ in the NRS pain score or VISA score compared to athletes with an initial symptom duration of 1–2 months or 2–3 months (Tran et al. 2020; Malmgaard‐Clausen et al. 2021). However, there is conflicting evidence on whether initial symptom duration plays a role in the improvement of symptoms over time. One study investigating early tendinopathy in recreational runners with AT found that after 12 weeks of exercise therapy, improvement in clinical outcomes (NRS pain level) was greater in athletes with shorter symptom duration (< 1 month) compared to those with longer symptom duration (1–2 months or 2–3 months) (Malmgaard‐Clausen et al. 2021). Another study investigated the effect of symptom duration on recovery in patients with early and late AT after 16 weeks of exercise therapy (Hanlon et al. 2023). Participants were categorised based on the initial symptom duration: < 3 months, 3–6 months, 6–12 months or > 12 months. The investigators observed no differences in VISA scores between the groups. Both studies (Malmgaard‐Clausen et al. 2021; Hanlon et al. 2023) found that all participants with early tendinopathy improved clinically (NRS and VISA) after 12–16 weeks of exercise therapy. In our study, all groups improved clinically after 1 year for most NRS pain scores and VISA score, indicating that elite athletes with early tendinopathy behave similarly to recreational athletes (Malmgaard‐Clausen et al. 2021; Hanlon et al. 2023). It should be noted that, in the present study, all symptom duration groups improved equally after 1 year, although the multiple regression analysis indicated that longer symptom duration was associated with greater reductions in pain during functional tests (Supporting Information S1: Table S6), whereas other studies have shown greater clinical improvement when the symptom duration was less than 3 months (Malmgaard‐Clausen et al. 2021). This could be due to our follow‐up taking place 1 year after baseline examination instead of 3 months (Malmgaard‐Clausen et al. 2021) or 16 weeks (Hanlon et al. 2023). It is possible that by waiting for 1 year before follow‐up, differences in the trajectories of the duration groups on the shorter term have evened out. However, data at the first follow‐up after 12 weeks in the present cohort (Meulengracht et al. 2024) did not indicate a difference in improvement dependent on the symptom duration.

Interestingly, the elite athletes with early AT and PT tendinopathy in our study exhibited clinical relevant improvements in symptoms (NRS pain scores) after 1 year, similar to previous loading‐based rehabilitation intervention studies in recreational athletes with chronic AT (Beyer et al. 2015) and PT (Agergaard et al. 2021). An important difference to these previous studies is that we did not prescribe any specific exercise therapy, the present elite athletes just received instructions on pain‐guided activity modification (Silbernagel et al. 2007; Silbernagel and Crossley 2015; Sprague et al. 2021) at baseline. It should be mentioned that it has been previously shown that loading‐based rehabilitation may not be as effective in elite athletes with chronic tendinopathy when sports activity is continued (Visnes et al. 2005; van Ark et al. 2016; Rieder et al. 2022). Many of our athletes reported engaging in some form of resistance training as a part of their rehabilitation, which might be due to their easier access to treatment and support from physiotherapists and coaches compared to recreational athletes. Although we did not supervise or control the quality and amount of resistance training, these elite athletes demonstrated similar clinical symptom improvement to previous training intervention studies. In the AT group, 74% (20/27) became asymptomatic (≥ 87 points in VISA score) and only 33% (9/27) did not achieve a relevant clinical improvement (≥ 13 points in VISA score) (Roos et al. 2004; Hernandez‐Sanchez et al. 2014; Murphy et al. 2018). In the PT group, 52% (13/25) became asymptomatic but only 32% (8/25) did not achieve a relevant clinical improvement. Approximately 2/3 of the elite athletes in the present study achieved a relevant clinical improvement, which is a better long‐term prognosis than what normally is seen in elite volleyball players (Visnes et al. 2024).

Earlier studies (mainly in recreational athletes) have shown that chronic tendinopathy is characterised by fluid accumulation and a change in tendon structure causing thickening of the tendon as well as increased blood vessel formation and blood flow to the tendon (Magnusson et al. 2010; Tran et al. 2020). Additionally, studies have reported reduced PD in response to training, both in the short term for PT (Kongsgaard et al. 2009) and in the long‐term for AT (Beyer et al. 2015). However, our study revealed a lower echogenicity for PT compared to the contralateral leg at baseline, but no differences in PD over time for PT and AT. Considering the high prevalence of bilateral tendinopathy, the lack of a difference between the symptomatic and contralateral tendon might indicate that the contralateral leg is in an early phase of overuse but without onset of symptoms. This is supported by other studies that have found that tendon pathology is detectable before any debut of symptoms occur (Cook, Khan, Kiss, Purdam, et al. 2000; Fredberg and Bolvig 2002; Malliaras and Cook 2006; Abate et al. 2009; Visnes et al. 2015; McAuliffe et al. 2016).

The peritendinous AP thickness of the AT decreased after 1 year, and the distal part of the tendinopathic Achilles tendon showed a tendency towards a decreased AP thickness. However, no changes were observed after 1 year in the patellar tendon in any of the parameters. Our findings could indicate that whereas thickening of tendon is a sign of developing tendinopathy, the improvement in clinical symptoms in the recovery period is not closely associated with a loss of this tendon thickening. It can be hypothesised that both the injured and the contralateral tendons of elite athletes may differ or might have adapted to higher levels of loading compared to those of recreational athletes. This could explain the absence of an increase in PD or hypoechogenicity when comparing the injured leg to the contralateral one, as the healthy leg in elite athletes may also have been subjected to excessive loads (Couppé et al. 2008, 2021). Finally, the presence of a hypoechoic region is typically subjectively evaluated by inspection, but our quantitative results did not demonstrate any difference between subgroups or over time in elite athletes with early tendinopathy. Others have also found that imaging outcomes (US and MRI) does not correlate with clinical outcomes, and therefore, imaging are not conclusive for prognosis of tendinopathy (Visnes et al. 2024).

We also observed in this study that a lower PD at baseline was predictive of lower AP thickness. This implies that a less favourable morphological status (indicated by higher PD levels) at baseline negatively affects the structural recovery of the tendon over a 1‐year period. Further, greater VISA score at baseline predicted a greater improvement (normalisation) in echo intensity. Together, these observations indicate that athletes with superior tendon health at baseline (lower PD and greater VISA score) experience greater improvements in tendon structure (reduced thickness and more normalised echo intensity) after 1 year. Lastly, we observed that the prolonged initial symptom duration predicted reductions in NRS pain scores during the functional tests after 1 year. It is worth noting that the interaction between symptom duration and time for this outcome was not statistically significant in the primary analysis, which may be because the regression model also included the baseline NRS values. Indeed, most of the regression models included a negative relation between baseline scores and the change in these same scores after 1 year.

A strength of this study is that a variety of different sports were represented, which makes the data more transferable to different sports disciplines, but it is also a limitation due to increased heterogeneity, which could shroud effects that are not universal to all sports. Another strength of our study is the 1‐year follow‐up period, but as discussed earlier, this might also have masked differences between the subgroups that may have occurred earlier. A limitation of this study is that it was an observational study, and we can therefore not be certain which interventions including the pain‐guided activity modification approach, the individual athlete may have undertaken (besides already noted) apart from the exclusion criteria. Further, since subgrouping was based on the clinical symptom duration, it cannot be excluded that athletes in T1 had experienced morphological changes for a longer period than those in T2 or T3. Furthermore, elite athletes may face economic or performance pressures that could lead them to push through pain, potentially reducing the efficacy of recommended pain‐guided activity modifications. Moreover, the contralateral tendon was used as a control, but it has been suggested that the asymptomatic (contralateral side) tendon may not be a true control as it may contain subclinical changes that could develop into symptoms later (Paavola et al. 2000; Docking et al. 2015; Rabello et al. 2020). Due to the observational nature of the study, it was not powered to detect a specific clinical change and, consequently, it may be underpowered to detect some meaningful differences, especially in subgroup analyses. Furthermore, recent studies have raised concerns regarding the validity of the VISA‐A (Comins et al. 2021) and VISA‐P (Agergaard et al. 2023) scores, which could have impacted the results of the present study.

Our findings show that elite athletes with early AT and PT improve their symptoms and function without necessarily changing morphology over the course of 1 year. Asymptomatic tendon pathology assessed by using ultrasound has in many studies demonstrated to be an important risk factor for developing tendinopathy (Khan et al. 1997; Cook, Khan, Kiss, Purdam, et al. 2000; Fredberg and Bolvig 2002; Visnes et al. 2015; McAuliffe et al. 2016). Therefore, future studies should elucidate whether such changes can be prevented and optimise treatment strategies if pathology is already present by for example, long‐term follow‐up studies. Furthermore, our multiple regression results indicate that early detection of a tendinopathy, whereas symptoms and pathology is minimal, could benefit subsequent recovery, since reduced baseline values was associated with greater long‐term improvement and recovery in both symptoms and morphology. This information seems valuable for the clinician to share with the athlete maximise future recovery. Further, our findings suggest a similar behaviour in clinical outcomes between recreational and elite athletes. This is important as it implies that exclusive inclusion of elite athletes may not be imperative for advancing tendinopathy research in this population. Another important perspective is that many adult athletes suffering from tendinopathy experience similar symptoms during adolescence (Cook, Khan, Kiss, and Griffiths 2000; Simpson et al. 2016). Consequently, we advocate that early tendinopathy should be further investigated in highly active children and adolescents to better understand its early manifestations and progression.

## 5 Conclusions

Our findings suggest that, in elite athletes, symptoms of both early AT and early PT improved clinically after 1 year but with very moderate (only in AT) changes in morphology through this period. Intriguingly, elite athletes appear similar in the presentation and response to tendinopathy in terms of clinical improvement when compared to previous studies in recreational athletes. Finally, less severe baseline symptoms in terms of lower PD activity and better VISA score indicate a better prognosis for both morphology and symptoms in elite athletes.

## Ethics Statement

The study conformed to the guidelines of The Declaration of Helsinki and was approved by the Regional Ethics Committee (H‐16019857) and was preregistered on ClinicalTrials.gov (identifier: NCT03642392) in August 2018.

## Consent

All participants signed a declaration of informed consent at the time of inclusion in the study.

## Conflicts of Interest

The authors declare no conflicts of interest.

## Supporting information

Supporting Information S1

## Data Availability

The data that support the findings of this study are available from the corresponding author upon request. The data are not publicly available due to privacy or ethical restrictions.
