# Supplementary material for: One‐Year Follow‐Up of Clinical and Morphological Outcomes in Elite Athletes With Early‐Stage Lower Extremity Tendinopathy
Source: Eur J Sport Sci. 2025 Apr 22;25(5):e12303. doi: 10.1002/ejsc.12303 (PMC12013731; doi:10.1002/ejsc.12303)
Supplement: Supplementary file 1 — Supporting Information S1 [file EJSC-25-e12303-s001.docx]

Supplementary material

US equipment settings and examination specification:
US examination of the tendons was performed using a HI VISION Ascendus ultrasound machine (Hitachi Medical Systems). Settings for Gray-scale (GS) and power doppler (PD) were identical for all examinations. The Grey-scale examination for the patellar tendon was performed with the participant in a seated position with 90 degrees of hip and knee flexion, and for Achilles tendons with the participant lying prone with their feet hanging off the bed while some pressure was applied to the foot to avoid slack on the tendon. The examination was performed with a long linear transducer (EUP-L53L, Hitachi Medical Systems), the depth fixed at 4.5 cm, a dynamic range of 70, and a gain of 20. The transducer was placed sagittally at a 90-degree angle and moved medially to laterally to find the location of the greatest anterior-posterior thickness of the tendon.

The Power Doppler (PD) examination for the patellar tendon was performed with participants in a supine position and with a stretched and relaxed knee. For the Achilles tendon, it was performed in a prone position with the feet hanging freely from the bed. PD imaging used a shorter linear transducer (EUP-L75, Hitachi Medical Systems), the depth fixed at 2.0 cm, a dynamic range of 70, a colour Doppler frequency of 10 MHz, a pulse repetition frequency of 250 Hz, and a colour gain of 37.

| Achilles | Baseline means ± SEM | 52 weeks means ± SEM | Delta | 95% CI | Delta (%) | P-value |
| --- | --- | --- | --- | --- | --- | --- |
| All hypoechoic region echogenicity % of contralateral | 96.67 ± 5.30 | 102.98 ± 4.92 | 6.23 | -16.21 to 3.76 | 6.44 | 0.206 |
| T1 | 97.70 ± 4.33 | 106.39 ± 12.44 | 9.30 | -32.41 to 13.80 | 9.52 | 0.659 |
| T2 | 105.29 ± 5.53 | 109.28 ± 8.66 | 3.63 | -22.53 to 15.27 | 3.45 | 0.944 |
| T3 | 87.02 ± 5.02 | 93.28 ± 8.48 | 5.75 | -28.58 to 17.07 | 6.61 | 0.886 |
| All hypoechoic region echogenicity % of whole injured tendon | 88.58 ± 1.96 | 85.35 ± 1.50 | -3.80 | -1.28 to 8.88 | -4.29 | 0.134 |
| T1 | 89.02 ± 3.06 | 83.99 ± 5.02 | -3.31 | -8.51 to 15.12 | -3.72 | 0.852 |
| T2 | 91.73 ± 3.35 | 88.34 ± 4.37 | -3.37 | -5.97 to 12.71 | -3.67 | 0.731 |
| T3 | 84.99 ± 5.88 | 83.71 ± 6.04 | -4.72 | -7.01 to 16.44 | -5.55 | 0.663 |
| All max thickness (mm) | 6.93 ± 0.19 | 6.63 ± 0.29 | -0.35 | -0.20 to 0.89 | -5.05 | 0.1954 |
| T1 | 6.56 ± 0.43 | 7.18 ± 1.01 | 0.42 | -1.60 to 0.84 | 6.40 | 0.7718 |
| T2 | 7.02 ± 0.31 | 6.20 ± 0.28 | -0.69 | -0.30 to 1.69 | -9.83 | 0.2292 |
| T3 | 7.20 ± 0.52 | 6.95 ± 0.42 | -0.77 | -0.48 to 2.20 | -10.70 | 0.3235 |
| All proximal thickness (mm) | 6.14 ± 0.13 | 5.89 ± 0.31 | -0.28 | -0.25 to 0.80 | -4.56 | 0.2791 |
| T1 | 5.89 ± 0.47 | 6.50 ± 1.01 | 0.43 | -1.6 to 0.79 | 7.31 | 0.7471 |
| T2 | 6.25 ± 0.30 | 5.52 ± 0.23 | -0.67 | -0.29 to 1.62 | -10.72 | 0.230 |
| T3 | 6.27 ± 0.49 | 5.65 ± 0.37 | -0.59 | -0.61 to 1.80 | -9.41 | 0.512 |
| All distal thickness (mm) | 6.26 ± 0.08 | 5.92 ± 0.26 | -0.44 | -0.07 to 0.95 | -7.03 | 0.089 |
| T1 | 6.14 ± 0.40 | 6.43 ± 0.89 | 0.07 | -1.26 to 1.12 | 1.14 | 0.998 |
| T2 | 6.42 ± 0.29 | 5.62 ± 0.24 | -0.72 | -0.21 to 1.66 | -11.21 | 0.1597 |
| T3 | 6.23 ± 0.46 | 5.70 ± 0.39 | -0.66 | -0.52 to 1.84 | -10.60 | 0.4033 |
| All peritendinous thickness (mm) | 8.32 ± 0.11 | 7.61 ± 0.18 | -0.79 | 0.15 to 1.43 | -9.50 | 0.0188* |
| T1 | 8.21 ± 0.64 | 7.46 ± 0.69 | -0.97 | -0.53 to 2.47 | -11.82 | 0.2858 |
| T2 | 8.20 ± 0.35 | 7.39 ± 0.24 | -0.72 | -0.46 to 1.89 | -8.78 | 0.3321 |
| T3 | 8.54 ± 0.66 | 7.97 ± 0.55 | -0.68 | -0.80 to 2.17 | -7.96 | 0.5642 |
| All power Doppler (mm^2^) | 4.96 ± 1.03 | 5.91 ± 4.62 | 0.68 | -6.80 to 5.44 | 13.78 | 0.818 |
| T1 | 7.02 ± 3.24 | 15.12 ± 13.59 | 7.44 | -22.06 to 4.33 | 107.79 | 0.467 |
| T2 | 3.75 ± 1.48 | 1.94 ± 0.94 | -1.65 | -8.93 to 12.24 | -44.12 | 0.970 |
| T3 | 4.11 ± 2.02 | 0.67 ± 0.25 | -3.86 | -10.39 to 18.11 | -94.00 | 0.866 |

*Table 4 - Ultrasonographic outcomes for Achilles tendinopathies*

*Means are of the raw data (including missing values) while Delta values and their associated statistics are based on the predicted means of the statistical model (accounting for missing values). * indicates statistical significance (p<0.05).
Abbreviations; SEM: Standard error of mean. CI: Confidence interval. T1: Initial symptom duration <1 month. T2: Initial symptom duration 1-2 months. T3: Initial symptom duration 2-3 months. All: refers to T1, T2 and T3 combined.*

| Patellar | Baseline means ± SEM | 52 weeks means ± SEM | Delta | 95% CI | Delta (%) | P-value |
| --- | --- | --- | --- | --- | --- | --- |
| All hypoechoic region echogenicity % of contralateral | 89.95 ± 4.47 | 92.05 ± 0.58 | 2.57 | -15.50 to 10.35 | 2.86 | 0.682 |
| T1 | 82.06 ± 8.48 | 92.41 ± 17.40 | 13.55 | -43.76 to 16.66 | 16.51 | 0.589 |
| T2 | 97.54 ± 9.75 | 92.83 ± 10.10 | -4.71 | -23.18 to 32.60 | -4.83 | 0.962 |
| T3 | 90.27 ± 5.76 | 90.91 ± 7.95 | -1.12 | -24.45 to 26.69 | -1.24 | 0.999 |
| All hypoechoic region echogenicity % of whole injured tendon | 70.32 ± 3.20 | 72.32 ± 2.14 | 1.59 | -7.77 to 4.58 | 2.26 | 0.596 |
| T1 | 63.93 ± 3.52 | 68.38 ± 6.18 | 5.98 | -20.51 to 8.55 | 9.35 | 0.652 |
| T2 | 73.78 ± 3.70 | 72.86 ± 5.48 | -0.92 | -12.31 to 14.14 | -1.25 | 0.997 |
| T3 | 73.26 ± 5.79 | 75.72 ± 4.05 | -0.33 | -11.91 to 12.48 | -0.45 | 0.9998 |
| All proximal thickness (mm) | 7.98 ± 0.50 | 7.84 ± 0.53 | -0.13 | -0.46 to 0.71 | -1.63 | 0.6464 |
| T1 | 8.85 ± 0.72 | 8.90 ± 0.96 | -0.23 | -1.15 to 1.61 | -2.60 | 0.9642 |
| T2 | 7.12 ± 0.41 | 7.30 ± 0.59 | 0.18 | -1.50 to 1.05 | 2.53 | 0.9757 |
| T3 | 7.99 ± 0.44 | 7.32 ± 0.53 | -0.34 | -0.81 to 1.48 | -4.26 | 0.3343 |
| All mid thickness (mm) | 5.64 ± 0.23 | 5.62 ± 0.27 | -0.03 | -0.31 to 0.37 | -0.53 | 0.8604 |
| T1 | 6.04 ± 0.38 | 5.97 ± 0.57 | -0.15 | -0.66 to 9536 | -2.48 | 0.9525 |
| T2 | 5.23 ± 0.27 | 5.08 ± 0.20 | -0.15 | -0.58 to 0.87 | -2.87 | 0.9374 |
| T3 | 5.66 ± 0.21 | 5.80 ± 0.33 | 0.21 | -0.88 to 0.46 | 3.71 | 0.8149 |
| All distal thickness (mm) | 5.36 ± 0.31 | 5.27 ± 0.24 | 0.05 | -0.36 to 0.25 | 0.93 | 0.7225 |
| T1 | 5.71 ± 0.43 | 5.56 ± 0.55 | 0.08 | -0.81 to 0.65 | 1.40 | 0.9897 |
| T2 | 4.73 ± 0.16 | 4.79 ± 0.25 | 0.06 | -0.70 to 0.58 | 1.27 | 0.9943 |
| T3 | 5.63 ± 0.38 | 5.46 ± 0.25 | 0.02 | -0.62 to 0.58 | 0.36 | 0.9997 |
| All power Doppler (mm^2^) | 13.08 ± 3.24 | 10.85 ± 3.72 | -0.57 | -3.75 to 4.90 | -4.37 | 0.785 |
| T1 | 21.07 ± 10.15 | 16.08 ± 4.11 | -0.44 | -9.73 to 10.60 | -2.08 | 0.999 |
| T2 | 11.51 ± 3.61 | 12.81 ± 5.44 | 0.83 | -1.03 to 8.66 | 7.19 | 0.994 |
| T3 | 6.65 ± 3.15 | 3.66 ± 1.52 | -2.11 | -6.25 to 10.46 | -31.70 | 0.888 |

*Table 5 - Ultrasonographic outcomes for patellar tendinopathies*

*Means are of the raw data (including missing values) while Delta values and their associated statistics are based on the predicted means of the statistical model (accounting for missing values).
Abbreviations; SEM: Standard error of mean. CI: Confidence interval. T1: Initial symptom duration <1 month. T2: Initial symptom duration 1-2 months. T3: Initial symptom duration 2-3 months. All: refers to T1, T2 and T3 combined.*

| All patients | R^2^ (*Semi-partial R^2^ for parameters*) | Parameter estimate | S.E. | P-value |
| --- | --- | --- | --- | --- |
| Delta Doppler (mm^2^) | NS | NS | NS | NS |
| Delta AP Thickness (mm) | 0.520 | - | - | <0.0001 |
| *Baseline doppler (mm^2^)* | 0.189 | 0.083 | 0.021 | 0.0004 |
| *Baseline thickness (mm)* | 0.506 | -0.732 | 0.114 | <0.0001 |
| Delta Echo intensity (%) | 0.259 | - | - | 0.0029 |
| *Baseline VISA score* | 0.089 | 0.254 | 0.117 | 0.0363 |
| *Baseline echo intensity (%)* | 0.220 | -0.396 | 0.116 | 0.0016 |
| Delta NRS functional test | 0.656 | - | - | <0.0001 |
| *Initial symptom duration (mo)* | 0.069 | -0.809 | 0.29038 | 0.0082 |
| *Baseline NRS functional test* | 0.650 | -0.898 | 0.105 | <0.0001 |
| Delta VISA score | 0.335 | - | - | <0.0001 |
| *Baseline VISA score* | 0.335 | -0.712 | 0.159 | <0.0001 |

*Table 6 - Secondary outcomes
Predictions using backwards elimination (outcomes in regular font, predictions in italic font). Predictors that were eliminated due to non-significance are not shown. Echo intensity is of the hypoechoic region relative to the whole tendon on the injured side in percent.*

*Abbreviations: NS: non-significant. S.E.: Standard error. AP: Anterior-posterior. VISA score: points on The Victorian Institute of Sports Assessment Questionnaire. NRS: Pain on a numerical rating scale (0-10) during the functional test. Mo: months.*
